# Savouring as an Intervention to Decrease Negative Affect in Anxious Mothers of Children with Autism and Neurotypical Children

**DOI:** 10.3390/brainsci11050652

**Published:** 2021-05-16

**Authors:** Ariel S. Pereira, Atiqah Azhari, Chloe A. Hong, Gerin E. Gaskin, Jessica L. Borelli, Gianluca Esposito

**Affiliations:** 1 Psychology Program, School of Social Sciences, Nanyang Technological University, Singapore 639798, Singapore; AR0001RA@e.ntu.edu.sg (A.S.P.); nura0066@e.ntu.edu.sg (A.A.); chloeamanda.hong@u.nus.edu (C.A.H.); 2 Division of Behavioral and Organizational Sciences, School of Social Science, Policy and Evaluation, Claremont Graduate University, Claremont, CA 91711, USA; gerin.gaskin@cgu.edu; 3 Department of Psychological Science, University of California, Irvine, CA 92697, USA; jessica.borelli@uci.edu; 4 Lee Kong Chian School of Medicine, Nanyang Technological University, Singapore 639798, Singapore; 5 Department of Psychology and Cognitive Science, University of Trento, 38068 Rovereto, Italy

**Keywords:** savouring, affect, anxiety, mothers, children with autism spectrum disorder

## Abstract

Savouring is an emotion regulation strategy and intervention that focuses on the process of attending, intensifying and prolonging positive experiences and positive affect associated with these memories. Personal savouring involves a reflection on positive memories that are specific to the individual and do not involve others. In contrast, relational savouring entails reflecting on instances when people were responsive to the needs of their significant others. Such interventions hold potential benefits in enhancing positive affect (PA) and reducing negative affect (NA) for both parents of children with autism spectrum disorder (ASD) and parents of neurotypical children. Adults with greater symptoms of generalised anxiety have been found to have less PA and more NA. However, no study has investigated the effects of a mother’s anxiety symptoms on the efficacy of savouring in enhancing PA and reducing NA. Thus, this paper combined personal and relational savouring to investigate whether savouring may enhance PA and reduce NA of a pooled sample of mothers of neurotypical children and mothers of children with ASD. 52 mothers of neurotypical children and 26 mothers of children with ASD aged 3–7 years old were given a series of questionnaires and randomly assigned to either relational savouring or personal savouring conditions. In relational savouring, mothers were asked to reflect upon a shared positive experience with their child while in the personal savouring condition, a personal positive experience was recalled. Across mothers of children with ASD and neurotypical children, findings suggest that savouring leads to a decrease in NA (*p* < 0.01) but not increases in PA. Similarly, mothers with higher levels of anxiety experience a greater decrease in NA (*p* < 0.001) compared to mothers with lower levels of anxiety post-savouring. This study proposes that a brief savouring intervention may be effective among mothers of preschoolers. As lower levels of negative affect is linked to healthier psychological well-being, mothers might be able to engage in more effective and warm parenting after savouring exercises, which would cultivate positive mother-child relationships that benefit their children in the long-term.

## 1. Introduction

In contemporary life, it is easy to let moments pass us by. Yet, the process of reliving and recollecting positive moments can reap benefits. Savouring, a psychological exercise first conceptualized within the field of positive psychology, is the process of attending, intensifying and prolonging positive experiences [1]. People can savor experiences that have occurred in the past, experiences that are occurring in the moment, or experiences that are anticipated to occur in the future [1]. Personal savouring involves reflecting on personal positive memories whereas relational savouring encompasses reflecting on positive moments one has had with other individuals [2]. Savouring has been associated with an array of positive psychological outcomes, such as lower levels of depression, greater life satisfaction, and positive emotion [3]. Past savouring interventions have found savouring to be efficacious among various populations including mothers, military spouses, and couples in long-distance relationships [4,5,6].

In recent years, there has been greater emphasis placed on addressing the positive aspects rather than the negative problems faced in parenting a child with autism spectrum disorder (ASD) [7]. ASD is classified as a neurodevelopmental disability, and characteristics of the disorder may include social deficits in communication, accompanied by repetitive behaviours and restricted interests. With communication posing a significant challenge, such deficits are more easily identifiable when children reach the age of social interaction and display marked deviation from the milestones of neurotypical peers [8], around the age of two [9]. Existing on a spectrum, persons with ASD can exhibit widely differing symptoms and require assistance ranging from support to very substantial support [10]. This diagnosis has become more prevalent, and has increased by up to four times in the recent decade [11,12].

Affect, an emotional response, can be broadly classified as positive (PA; positive affect) or negative (NA; negative affect), and can be measured using the Positive Affect and Negative Affect Schedule (PANAS) scale [13]. The PANAS is an established self-report measure of PA and NA [13], designed to measure affect in various contexts such as at the present moment, the past day, week, or year. Furthermore, it is based on a two-dimensional conceptual model of mood [14], which proposes that PA and NA are seen as unique and independent constructs that are capable of being experienced simultaneously [15]. Studies generally find that compared to mothers of neurotypical children, mothers of children with ASD experience significantly less PA and more NA [16,17,18]. Several studies emphasise that mothers of children with developmental disabilities are more likely to experience NA compared to counterparts with neurotypical children [19,20,21], and this NA emerges in the child’s early years, from the time a child is diagnosed, and made more pronounced in the child’s later years when the child fails to hit crucial developmental milestones [22,23].

Brief savouring interventions have successfully improved PA and decreased NA [24,25]. Positive affect can be highly adaptive (review in [26]), serving as a psychological buffer by enabling mothers of children with ASD to cope during difficult situations [27,28]. Thus far, only one study has examined the effects of savoring on the PA and NA of mothers of neurotypical infants and toddlers, but not in mothers of children with ASD. In a seminal study on 435 parents of neurotypical children, [6] assessed a subsample of 64 parents and found that both personal and relational savoring conditions elicited heightened PA and reduced NA. Correspondingly, savouring interventions may be promising in their ability to improve well-being among parents. Since mothers are generally the primary caregivers of children with autism and face an increased risk of adverse psychological outcomes such as heightened anxiety [29,30,31,32], our current study intends to build and extend on the findings of [6] to mothers of neurotypical children and mothers of children with ASD.

The psychological profile of mothers of children with ASD greatly differs from that of mothers of typical children, with the former experiencing significantly higher levels of anxiety symptoms (e.g., [29,33,34,35]). General anxiety levels is characterised by excessive anxiety and worry about various events or activities. Over half of parents of children with ASD experience severe generalized anxiety [36] which may stem from worries about confronting their child’s problematic and inappropriate behaviours [34,37,38], managing shortfalls in time and monetary resources [29,31] and overcoming the emotional turmoil they experience such as guilt and anger on a daily basis [30,32,36]. As PA is found to be lacking among people with anxiety disorders [39], highly anxious mothers may also stand to gain from savoring interventions.

Since the study by [6] showed that personal and relational savouring both efficaciously improved mothers’ affect, the two savoring conditions would be combined in this study. The broader literature on savouring likewise points to the efficacy of either types of savoring in enhancing emotional affect (e.g., [1]). Subsequently, we embarked on this study with four hypotheses. First, similar to the findings in the study by [6], we hypothesize that a decrease in Positive and Negative Affect Schedule—Negative Affect (PANAS-NA) will be seen post-savouring. Secondly, we hypothesize that savouring will increase Positive and Negative Affect Schedule—Positive Affect (PANAS-PA) among mothers of young children due to enhanced positive perceptions, similar to the success of other positive interventions in literature as found above. In the event where PANAS-NA was reduced after savouring, or PANAS-PA was enhanced post-savouring, we embark on an exploratory hypothesis to investigate whether mothers’ generalised anxiety is associated with change in affect.

## 2. Materials and Methods

### 2.1. Participants

This study obtained approval from NTU Institutional Review Board- Social Behavioural Educational Sciences (IRB; REF number: 2017-01-032) (CRC; REF number 612-2018). Participants were informed that participation was entirely voluntary and that they could withdraw at any point of time in the study. During the pre-screening call, participants were provided with the details of the study and gave their consent both during the call and before they proceeded with the online survey.

To participate in this study, mothers in both groups were required to be (1) proficient in English as the study was conducted in English, (2) currently residing in Singapore (3) above 21 years old, (4) have a target child between the ages of 3–7 years old at the time of the survey, and (5) possess the ability to read and understand English proficiently. Furthermore, mothers in both groups should have no (1) known mental disorders and (2) severely impaired cognitive deficits that may prevent them from understanding and responding to the online questionnaires. The minimum age of three years old was incorporated as an inclusion criterion for the Singaporean context of the study as a previous Singaporean study by [40] has shown that most children in Singapore are formally diagnosed at the age of three. Moreover, the target child in the ASD group should have an official ASD diagnosis. In contrast, the target child in the control group should not have any known diagnosis. This study was distributed via advertisements on online platforms, requesting for mothers to participate in a study focusing on mother-child relationships. Some early intervention centres in Singapore also helped disseminate this information.

**Figure 1:**
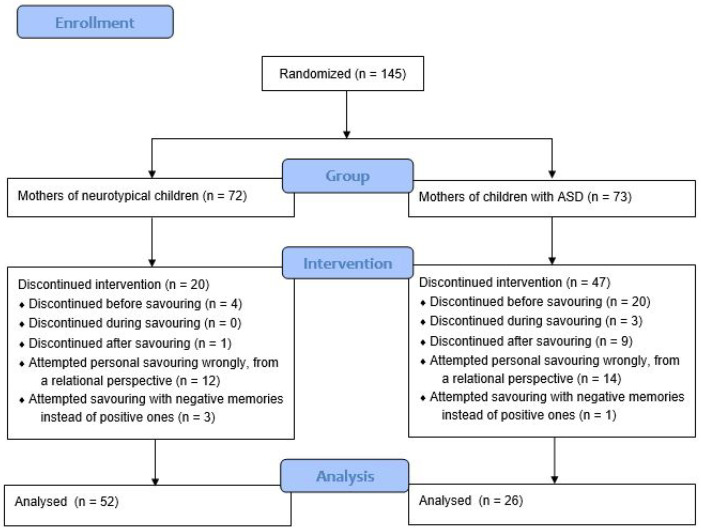
CONSORT DPI: Figure moved after the paragraph where it is first mentioned. Please confirm. Please add space around = in the picture.Flow Chart depicting the reasons for removal of incomplete data in both mothers of neurotypical children and children with ASD.

Over the span of two years, 72 mothers of neurotypical children and over the span of three years, 73 mothers of children diagnosed with ASD participated in this study. There was a high rate of attrition among eligible participants, especially among mothers of children with ASD. Out of which, 52 mothers of neurotypical children completed the entire study (73.61% completion rate), while 26 mothers of children with ASD completed the study (35.62% completion rate). Mothers of children in both groups who included relations of others in their personal savouring were excluded from this analysis. The eventual sample in each group consisted of 52 mothers of neurotypical children and 26 mothers of children with ASD (N = 78). The reasons for removal of incomplete data have been reported in the CONSORT flow chart in Figure 1. In the ASD group, 26 mothers between the ages of 26 to 47 (M = 35.73, SD = 4.84) of children who have been diagnosed with ASD between the ages of 3 to 7 (M = 5.44, SD = 1.19) were in this group. The control group consisted of 52 mothers between the ages of 22 years to 44 years old (M = 35.12, SD = 4.34) of neurotypical children between the ages of 3 to 6 (M = 4.10, SD = 1.19).

### 2.2. Procedure

A pre-screening call was made to interested participants to determine if they fit the criteria required for the study. Subjects were strongly encouraged to complete the study in one sitting, within the span of one week. Eligible participants were provided with a confirmation email link to the survey with their own unique study code. A brief explanation of savouring was given and consent from participants was sought prior to the administration of the study. The data was administered via an online data collection tool, Qualtrics (Qualtrics, Provo, UT, USA). In addition to other scales, baseline measurements for PANAS [13] were taken before the random assignment of the experimental condition (of personal savouring or relational savouring) was administered. Participants in the personal savouring condition took an average of 147 s to submit the page on the savouring task while those in the relational savouring condition took an average of 118 s.The PANAS scale was re-administered to mothers in both conditions after the experimental savouring condition, and generalised anxiety [41,42]) was measured after the administration of savouring intervention. Demographic information of mothers was recorded in the last section of the survey. Upon completion of the study, a debriefing form was sent to participants, and they were given monetary compensation as a token of appreciation.

### 2.3. Measures

#### 2.3.1. Generalised Anxiety Disorder-7 (GAD-7)

The GAD-7 scale [41,42] is a 7-item self-report questionnaire that measures the degree of worry and anxiety. Items including, “Worrying too much about different things,” are rated on a response scale from 1 (not at all) to 3 (nearly every day). Items were then added to constitute a total score (range 0–21), in which higher scores represent greater severity of anxiety. The scale has been validated in 2740 primary care patients [41] and is demonstrated to have good sensitivity and specificity as a screener for GAD, panic, social anxiety, and posttraumatic stress disorder [42]. It has been proven to have excellent internal consistency (α = 0.92), good test-retest reliability (intraclass correlation = 0.83) and strong criterion validity. Convergent validity was seen from the correlations with 2 anxiety measures: the Beck Anxiety Inventory (r = 0.72) and the anxiety subscale of the Symptom Checklist-90 (r = 0.74; [41]). The Cronbach’s alpha for the sample in this study was high at 0.95.

#### 2.3.2. Positive Affect and Negative Affect Schedule (PANAS)

Mood was measured using the PANAS scale, an established self-report measure of PA and NA [13]. Mothers answered 20 questions from the general dimension of the PANAS-SF scale [43], which consisted of ten items from the general dimension of PA and 10 items which reflect negative moods as general PA and NA was our interest in this study. The PANAS scale was administered immediately preceding and succeeding the savouring condition. For each item, mothers were instructed to report their feelings on a scale of 1–5 (1 ‘very slightly or not at all’ to 5 ‘extremely’). PA and NA are considered distinct from each other [13]. This measure has high validity with a Cronbach alpha coefficient of 0.89 to 0.85 respectively for PA and NA, and the test-retest correlation scores for both PA and NA respectively was 0.54 and 0.45 [13]. The scores for these corresponding 10 items of PA and NA were summed up pre and post-test and the changes in PA and NA were also tabulated. In this study, the Cronbach’S alpha coefficient was 0.97 for PA and 0.96 for NA.

### 2.4. Experimental Condition

Through random assignment, mothers in the ASD and control group were placed into one of two different savouring conditions—relational and personal savouring. The tasks in these interventions involve mental reflection and are similar to the tasks in [6] and were developed in consultation with the senior author on that study.

#### 2.4.1. Relational Savouring

The relational savouring task prompted participants to reflect on an occasion where they felt connected, close to or in tune with their child, regardless if it was a significant milestone or a daily affair. Participants could proceed to the next page after a minute. Follow up questions involved guiding mothers to describe the positive experience and the details surrounding this event. Mothers were then encouraged to utilise the next two minutes to let their mind wander and place their attention on positive aspects of their chosen event (see Table A1 in Appendix A).

#### 2.4.2. Personal Savouring

Identical to the relational savouring task, the only difference in the personal savouring condition was to exclude the presence of another in their savouring event. Mothers were therefore prompted to spend a minute reflecting on an occasion where they felt a positive and personal experience where no one else was present, it could potentially be a time where they felt a sense of achievement (see Table A2 in Appendix A). Examples of the prompt include asking them to come up with a memory of a recent time when you have felt happy. This should be something you experienced on your own (without anybody else present) and something you enjoyed but haven’t had time to really think about.” Other questions included “How did you feel at the time?” and “What thoughts did you have at the time?”

### 2.5. Analytical Plan

Sociodemographic information and descriptive statistics of mother’s age and child’s age would be reported for the two samples of mothers. Preliminary analyses on the types of pronouns participants used in the savouring task were conducted to determine if personal and relational savouring conditions significantly differed from each other. First-person singular pronouns (“I” and “me”) and first-person plural pronouns (“us” and “we”) and (“he”, “she”, “him” and “her”) were extracted from participants’ response to the savouring task, for personal and relational savouring, respectively. Chi-square tests were conducted on the frequency of pronoun category usage across the two savouring conditions. Inferential analyses addressing the three hypotheses were subsequently conducted. To test the first hypothesis, that savouring would lead to a decrease in PANAS-NA, a one-sided paired Wilxoxon signed rank test was conducted, comparing PANAS-NA post-savouring to pre-savouring levels. To test the second hypothesis, that savouring would increase PANAS-PA, a one-sided paired Wilxoxon signed rank test was also conducted, contrasting PANAS-PA post-savouring to pre-savouring scores. As there were two models to be tested, Bonferroni correction was applied onto alpha, such that alpha = 0.05/2 = 0.025. To test the third exploratory hypothesis, that higher levels of generalised anxiety in the mother would result in a greater decrease in PANAS-NA, a nonparametric Kendall–Theil linear regression test was conducted where change in PANAS-NA (PANAS-NA_Δ_; Post-Pre) was the dependent variable, and generalised anxiety score was the independent factor.

## 3. Results

Sociodemographic information, and descriptive statistics of mother’s age and child’s age for the two samples of mothers are reported in Table 1.

### 3.1. Preliminary Analyses

#### 3.1.1. Between Savouring Conditions

A preliminary analysis was conducted to determine if there were significant differences in the use of pronouns across personal and relational savouring conditions. The personal savouring condition should have more first-person singular pronouns (“I” and “me”) pronouns since this condition focuses on the participant only, whereas the relational savouring condition should contain more plural pronouns (“us” and “we”) and (“he”, “she”, “him” and “her”) since it encompasses the participant and another individual. A chi-square test was conducted to compare the average usage of first-person singular pronouns (“I” and “me”) and that of first-person plural pronouns (“us” and “we”) and (“he”, “she”, “him” and “her”) respectively for each savouring question. However, no significant finding emerged between the use of singular and plural pronouns across the two conditions which suggested that participants did not engage in fundamentally differently savouring experiences across conditions (see Table A3 and Table A4 in Appendix A). Since the two savouring conditions did not differ in the preliminary analyses, we decided to pool the two conditions together for the subsequent analyses.

#### 3.1.2. Between Groups of Mothers

**Figure 2:**
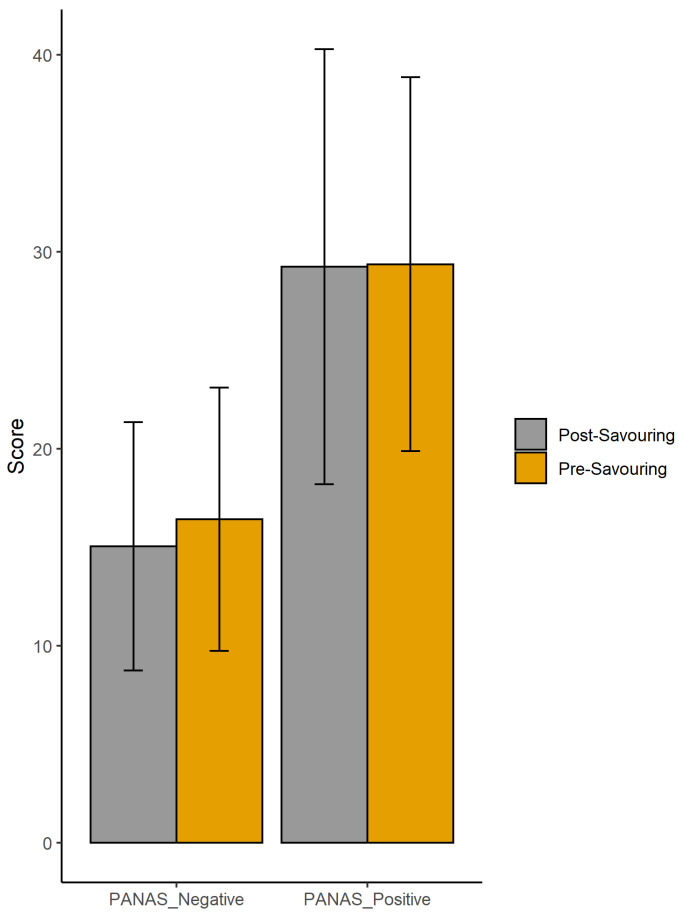
Bar plot of PANAS-Negative and PANAS-Positive pre- and post- savouring.

Mean and standard deviation values of PA and NA for groups of mothers are reported in Table 2. To determine if there were significant differences across the two groups of mothers, a two-sample nonparametric Mann-Whitney test was conducted between the two groups for PANAS-PA_pre_, PANAS-PA_pos_, PANAS-NA_pre_ and PANAS-NA_pos_ values (see Table 2). Since no significant difference was found between the PA and NA values of the two groups of mothers, we decided to pool the two diagnostic groups together for the subsequent analyses. Figure 2 shows pooled mothers’ PA and NA scores before and after savouring conditions.

### 3.2. Inferential Analyses

Since there was no significant difference in pronoun usage between savouring conditions, and no significant difference between the means of PA and NA between groups of mothers, inferential analyses were conducted on a pooled sample of mothers and savouring conditions. Shapiro-Wilk normality test was conducted on the pooled sample which found the pooled sample to be non-normal (W = 0.846, *p* = 1.625 × 10^−7^). Subsequently, nonparametric tests were used for the inferential analyses.

To test hypothesis 1, a one-sided paired dependent 2-group paired Wilcoxon signed rank test was conducted between PANAS-NA_pos_ and PANAS-NA_pre_ for each of the two groups of mothers, and for the pooled group of mothers (see Table 3), where PANAS-NA_pos_ was expected to be less than PANAS-NA_pre_. Post savouring, PANAS-NA_pos_ was found to be significantly less than PANAS-NA_pre_ for mothers of typical children (V = 274, *p* = 0.013) and for the pooled group of mothers (V = 616, *p* = 0.0036).

**Figure 3:**
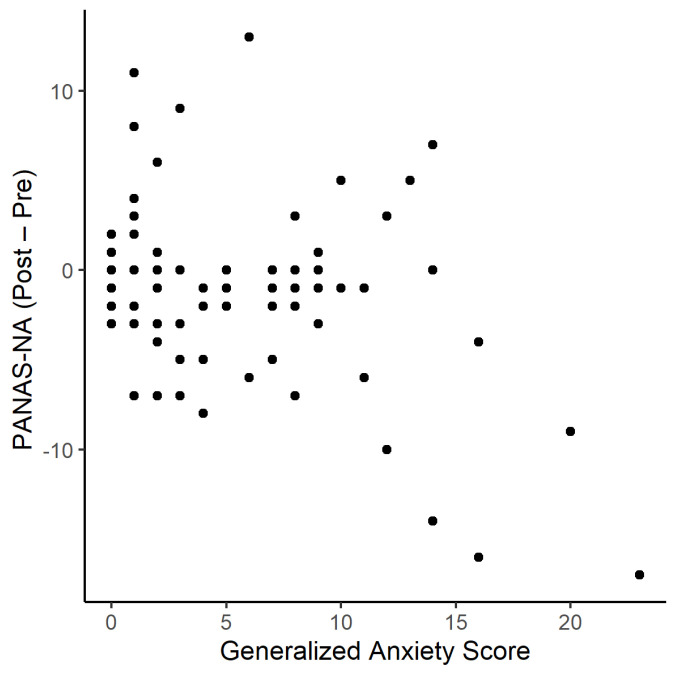
Scatter plot of Generalized Anxiety score against PANAS-NA_Δ_(Post−Pre).

Subsequently, the nonparametric Kendall–Theil linear regression test was conducted to determine the effect of generalised anxiety on PANAS-NA_Δ_ for the pooled group of mothers. The linear regression test revealed a significant effect of generalised anxiety on PANAS-NA_Δ_ in the pooled group of mothers (V = 449.5, *p* = 0.007, 95% CI (−0.375, −0.05), see Figure 3).

To test hypothesis 2, a one-sided paired dependent 2-group paired Wilcoxon signed rank test was conducted between PANAS-PA_pos_ and PANAS-PA_pre_ for each of the two groups of mothers, and for the pooled group of mothers (see Table 3), where PANAS-PA_pos_ was expected to be greater than PANAS-PA_pre_. However, no significant difference emerged between mothers’ reported positive affect before and after savouring.

## 4. Discussion

This study aimed to examine whether savouring increased positive affect and/or reduced negative affect among mothers of typical and ASD children. A secondary aim of this study was to examine if general anxiety levels influence the efficacy of a brief savouring in mothers. Supporting the first hypothesis and congruent with past literature on savouring among mothers of infants [6], this study found that there was a decrease in NA after savouring among mothers of young children in both the ASD and control groups. However, contrary to the second hypothesis, savouring failed to significantly increase PA among mothers across both ASD and control groups after this brief online savouring intervention was administered. This study further demonstrated in an exploratory analysis that a greater decrease in NA was observed after savouring among mothers with higher levels of generalised anxiety.

In line with literature on savouring [6], both personal savouring and relational savouring resulted in significantly less NA in parents of typically developing children in comparison to controls. Moreover, this study provides support for the effectiveness of positive interventions in alleviating negative affect of people with high levels of generalised anxiety [44]. Lower maternal self-efficacy has been found to be correlated to higher self-reported maternal anxiety [45]. The process of savouring could have encouraged mothers to remember and relish their past achievements, focusing on moments whereby mothers have been successful in protecting, supporting or aiding in their child’s growth salient to mothers. This may, in turn, increase the self-efficacy of mothers, and hence, reduce their NA [46].

A decrease in NA experienced by mothers with higher levels of anxiety is also consistent with another study done on another positive psychology concept-mindfulness. Among those with high anxiety sensitivity but not low anxiety sensitivity, trait mindfulness was significantly related to a reduced NA when there was a brief stress inducement [47]. Given that savouring overlaps with mindfulness to the extent that it demands for people to be mindful of a particular experience [1], a savouring intervention may similarly activate the same mechanisms to reduce NA. There could also be a floor effect of NA among mothers with already low levels of anxiety, such that mothers who are higher in anxiety also have higher levels of NA. Therefore, partaking in savouring tasks would be more likely to be able to reduce already comparatively high levels of NA among mothers, compared to mothers with lower levels of anxiety with potentially already lower levels of NA at baseline.

There are some caveats which should be taken into consideration when interpreting the results of this study. First, a control group that was present in the [6] study was missing in the present study. The presence of a control group where neither the mothers of neurotypical children nor the mothers of children with ASD experienced savouring would have allowed us to rule out extraneous factors not directly related to the savouring task, such as mothers obtaining time to themselves when using the phone to complete the online intervention, that could have reduced negative affect. Future studies should include a control condition to strengthen the argument for the efficacious effects of savouring. Second, the savouring intervention was held for only one session, and no follow-up assessment was conducted to evaluate how long the effect of the intervention lasts for. Since mothers of both neurotypical children and children with ASD may experience recurring anxiety on a daily basis, future studies should incorporate follow-up interventions and assessments, such as a follow-up of the reduced effects of NA one month later, to investigate the frequency with which savouring needs to be practised to produce long-term psychological wellbeing outcomes. Third, parents were also encouraged to complete the survey in one sitting; however, there were some parents who completed the PANAS before and after the savouring intervention over one week. It is possible that maturation effects could have occurred due to other situations or experiences that took place during this interval, affecting the pre- or post- PANAS scores if they were not completed immediately before and after the savouring activity. Fourth, there is also a high attrition rate of mothers who did not complete this study, and as such, their responses were removed from the analysis. It is, however, unclear why they did not continue with the study. A possibility could be that mothers who dropped out may have children with more severe needs, thus they may not be able to afford the time to complete the study. However, this bias may also be reflective of mothers who are keen on understanding and improving the relationship with their child. Upon conducting Mann-Whitney U Tests and Fisher’s exact tests to determine if there were demographic differences between parents who did not complete the study and those who did, it was found that there were no significant differences between the two groups across all demographic variables (*p* > 0.05). Fifth, although the environmental context where mothers were doing the online intervention was not controlled for, this may increase the ecological validity of our study as in an online intervention, the environmental context may vary across mothers. Future studies may extend on this research to consider the environment of the mother as a factor that may enhance PA and NA. Moreover, this study may include a randomised control group in future to determine causality of savouring in future.

This current study adds to a body of literature that savouring may be especially useful to enhance affect among mothers by decreasing their NA. Mothers with anxiety may be able to harness these benefits. Mothers of preschool-aged children may stand to decrease negative feelings by savouring past experiences of achievements that had occurred both with and without their child. This is more so among mothers who experience greater levels of anxiety. Savouring may, therefore, be a possible intervention incorporated as an emotional coping strategy, perhaps in conjunction with other problem-solving strategies among mothers and especially those with anxiety to decrease NA. In problem-coping strategies (methods which tackle the issue or alter the source of stress), the relationship between affect and learning is complex and thus, high levels of NA may hinder a person’s ability to learn [48]. However, such strategies are still likely to play an important role in helping mothers cope [49]. Therefore, to enhance mothers’ receptivity to these strategies, incorporating elements of emotion coping that target managing affect (such as this savouring intervention) to decrease NA, at least temporarily, may be beneficial, especially among mothers with higher levels of anxiety. Moreover, lower levels of NA is linked to better psychological health. Therefore, savouring as an intervention may be helpful to improve the mental well being of mothers of young children with ASD and neurotypical children alike.

## Figures and Tables

**Table 1:** Sociodemographic information of participants.

	Sample 1: Mothers with Typical Children	Sample 2: Mothers with ASD Children
N	52	26
Savouring Condition	Personal: 22 Relational: 30	Personal: 10 Relational: 16
Age (Mothers)	Mean age of 35.1 years (SD = 4.34)	Mean age of 35.7 years (SD = 4.84)
Age (Children)	Mean age of 4.1 years (SD = 1.2)	Mean age of 5.4 years (SD = 1.2)
Sex (Children)	Female: 18 Male: 33	Female: 5 Male: 21
Education (Mothers)	4 GCE ‘O’ Levels 10 Diploma 38 Graduate Degree	3 GCE ‘O’ Levels School 7 Diploma 16 Graduate Degree
Employment Status	26 Employed Full-time 5 Employed Part-time 3 Self-employed 18 Unemployed	8 Employed Full-time 2 Employed Part-time 1 Self-employed 15 Unemployed
Marital Status	2 Unmarried 50 Married	26 Married
Household Income	10 $1000–$4999 21 $5000–$8999 11 $9000–$12,999 5 $13,000–$19,999 2 > $20,000	9 $1000–$4999 9 $5000–$8999 6 $9000–$12,999 1 $13,000–$19,999 1 > $20,000

**Table 2:** Mean, standard deviations and Mann Whitney U Test of pre-savouring positive affect (PANAS-PA_pre_), post-savouring positive affect (PANAS-PA_pos_), pre-savouring negative affect (PANAS-NA_pre_) and post-savouring negative affect (PANAS-NA_pos_) for mothers of typical children and children with ASD.

	ASD		Typical		Mann-Whitney Test	
	**Mean**	**SD**	**Mean**	**SD**	**U**	***p*** **-Value**
PANAS-PA_pre_	31.2	9.8	28	9.28	771	0.316
PANAS-PA_pos_	29.0	10.9	29	11	666.5	0.924
PANAS-NA_pre_	18.4	8.8	15	5.2	771.5	0.311
PANAS-NA_pos_	16.4	7.8	14	5.4	760	0.370

**Table 3:** Dependent 2-group paired Wilcoxon signed rank tests between pre-PA and post-PA, and pre-NA and post-NA for mothers of typical children, mothers of children with ASD and pooled mothers.

Group	N	Paired Comparisons	Paired Wilcoxon Test	
			**V**	***p*** **-Value**
ASD	26	PANAS-PA_pos_–PANAS-PA_pre_	46.5	0.996
		PANAS-NA_pos_–PANAS-NA_pre_	71	0.0621
Typical	52	PANAS-PA_pos_–PANAS-PA_pre_	471	0.406
		PANAS-NA_pos_–PANAS-NA_pre_	274	0.0132 *
Pooled	78	PANAS-PA_pos_–PANAS-PA_pre_	857.5	0.89
		PANAS-NA_pos_–PANAS-NA_pre_	616	0.00359 **

Note: * = *p* < 0.05, ** = *p* < 0.01.

## Data Availability

The data presented in this study are openly available in DR-NTU at https://doi.org/10.21979/N9/EXRKPJ, accessed on 14 May 2021.

## Appendix A

**Table A1:** Relational savouring memory prompt.

Parents of young children often tell us they don’t have much time to focus on positive experiences they’ve had with their children because they are so busy and don’t have time to stop and reflect. For this task, we would like you to come up with a memory of a time when you felt extremely connected, close, or “in-sync” with your child. We are especially interested in hearing about a time when you found joy in helping your child grow, or a time when your child needed you and you were there for your child. It may be a time when you felt like you comforted, soothed, protected, or supported your child. Feel free to choose something that you felt was a milestone or something simple that happens on a daily basis. Please spend one minute focusing on this memory. You will be asked some questions about the details of this event in the following section.
1. Using as much detail as possible, describe what happened. Please enter a minimum of 100 characters.
2. What was the air like? What was the weather like? Please enter a minimum of 20 characters.
3. What time of day did the moment occur? Please enter a minimum of 20 characters.
4. What were you wearing? Please enter a minimum of 20 characters.
5. What was your child wearing? Please enter a minimum of 20 characters.
6. How did you feel at the time? (excited, proud, calm, relaxed etc.) Please enter a minimum of 100 characters.
7. What thoughts did you have at the time? About your child? About your relationship? Please enter a minimum of 100 characters.
8. What thoughts are you having now about your child and about your relationship? Please enter a minimum of 100 characters.
Mindfulness prompt: For the next two minutes, please let your mind wander in any way you’d like related to this event. You may want to think about things you were asked about earlier or you may want to think about how this memory is related to your other relationships and your life. It’s normal for your mind to wander to other topics or feelings—if you notice that your mind has wandered, just gently bring it back to the positive aspects of this event. Let your mind wander in any way you’d like, but try to keep focused on the positive parts of this memory.

**Table A2:** Personal savouring memory prompt.

Parents of young children often tell us they don’t have much time to focus on themselves because they are so busy taking care of their children. The goal of this task is to help you focus on something positive that happened in your life lately. What I’d like you to do is to come up with a memory of a recent time when you have felt happy. This should be something you experienced on your own (without anybody else present) and something you enjoyed but haven’t had time to really think about. The memory should also be something you would like to spend some time thinking about. It can be something as simple as enjoying a good meal, or taking a nice walk. It could also be something as major as getting a promotion or accomplishing a big task. The important thing is to come up with a time when you were feeling happy, content, or relaxed. Please spend one minute focusing on this memory. You will be asked some questions about the details of this event in the following section.
1. Using as much detail as possible, describe what happened. Please enter a minimum of 100 characters.
2. What was the air like? What was the weather like? Please enter a minimum of 20 characters.
3. What time of day did the moment occur? Please enter a minimum of 20 characters.
4. What were you wearing? Please enter a minimum of 20 characters.
5. How did you feel at the time? (excited, proud, calm, relaxed etc.) Please enter a minimum of 100 characters.
6. What thoughts did you have at the time? Please enter a minimum of 100 characters.
7. What thoughts are you having now? Please enter a minimum of 100 characters.
Mindfulness prompt: For the next two minutes, please let your mind wander in any way you’d like related to this event. You may want to think about things you were asked about earlier or you may want to think about how this memory is related to your other relationships and your life. It’s normal for your mind to wander to other topics or feelings - if you notice that your mind has wandered, just gently bring it back to the positive aspects of this event. Let your mind wander in any way you’d like, but try to keep focused on the positive parts of this memory.

**Table A3:** Chi-square tests comparing the average usage of first-person singular pronouns (“I” and “me”) and of first-person plural pronouns (“us” and “we”) for each savouring question.

Savouring Question	X2	*p*
What happened?	3.07	0.38
Weather?	0.37	0.95
Time of day?	0.45	0.93
Wearing?	1.08	0.78
Feeling?	3.22	0.36
Thoughts then?	3.35	0.34
Thoughts now?	3.53	0.32

**Table A4:** Chi-square tests comparing the average usage of first-person singular pronouns (“I” and “me”) and of first-person plural pronouns (“he”, “she”, “him” and “her”) for each savouring question.

Savouring Question	X2	*p*
What happened?	2.73	0.44
Weather?	0.31	0.96
Time of day?	0.37	0.95
Wearing?	1.08	0.78
Feeling?	3.22	0.36
Thoughts then?	3.35	0.34
Thoughts now?	2.64	0.45
